# Complexity analyses show two distinct types of nonlinear dynamics in short heart period variability recordings

**DOI:** 10.3389/fphys.2015.00071

**Published:** 2015-03-10

**Authors:** Alberto Porta, Vlasta Bari, Andrea Marchi, Beatrice De Maria, Dirk Cysarz, Peter Van Leeuwen, Anielle C. M. Takahashi, Aparecida M. Catai, Tomaso Gnecchi-Ruscone

**Affiliations:** ^1^Laboratory of Complex System Modeling, Department of Biomedical Sciences for Health, University of MilanMilan, Italy; ^2^IRCCS Galeazzi Orthopedic InstituteMilan, Italy; ^3^Department of Cardiothoracic, Vascular Anesthesia and Intensive Care, IRCCS Policlinico San DonatoMilan, Italy; ^4^Department of Anesthesia and Intensive Care Unit, Humanitas Clinical and Research CenterRozzano, Italy; ^5^IRCCS Fondazione Salvatore MaugeriMilan, Italy; ^6^Integrated Curriculum for Anthroposophic Medicine, University of Witten/HerdeckeWitten, Germany; ^7^Department of Medicine, Institute for Integrative Medicine, University of Witten/HerdeckeHerdecke, Germany; ^8^Department of Biomagnetism, Grönemeyer Institute for Microtherapy, University of Witten/HerdeckeBochum, Germany; ^9^Research Laboratory in Health Elderly, Department of Physiotherapy, Federal University of São CarlosSão Carlos, Brazil; ^10^Cardiovascular Physiotherapy Laboratory, Department of Physiotherapy, Nucleus of Research in Physical Exercise, Federal University of São CarlosSão Carlos, Brazil; ^11^Department of Cardiology, S.L. Mandic HospitalMerate, Italy

**Keywords:** time irreversibility, local prediction, multiscale analysis, head-up tilt, gestational age, heart rate variability, autonomic nervous system, cardiovascular control

## Abstract

Two diverse complexity metrics quantifying time irreversibility and local prediction, in connection with a surrogate data approach, were utilized to detect nonlinear dynamics in short heart period (HP) variability series recorded in fetuses, as a function of the gestational period, and in healthy humans, as a function of the magnitude of the orthostatic challenge. The metrics indicated the presence of two distinct types of nonlinear HP dynamics characterized by diverse ranges of time scales. These findings stress the need to render more specific the analysis of nonlinear components of HP dynamics by accounting for different temporal scales.

## Introduction

The beat-to-beat changes of heart period (HP) about its mean value, usually referred to as HP variability, were originally described as a linear superposition of rhythms (Akselrod et al., 1981). This mathematical description has produced remarkable results by providing the basis for the computation of frequency domain indexes and their relation with autonomic nervous system activity (Task Force, 1996). Nonetheless, given that the cardiac control is carried out by multiple interacting regulatory mechanisms exhibiting relevant nonlinearities (Koepchen, 1991), the mere description of HP variability in terms of linear superposition of rhythms might be extremely limiting and disregard significant dynamical features. Therefore, since the seminal study by Akselrod et al. (1981) a variety of nonlinear methods has been devised to extract information from short HP variability series (Maestri et al., 2007). In spite of indubitable utility of nonlinear indexes (Voss et al., 1996; Wessel et al., 2000; Maestri et al., 2007), the presence of nonlinear dynamics in short HP variability recordings is controversial. Indeed, some studies associated nonlinear components of HP variability to the presence of a dominant respiratory sinus arrhythmia (Porta et al., 2000, 2007a) and to the action of inputs forcing the cardiovascular system (e.g., respiration) (Fortrat et al., 1997; Kanters et al., 1997; Porta et al., 2007a). Conversely, other studies found that the incidence of nonlinear dynamics are not affected by the magnitude of the orthostatic challenge being relevant at rest, thus suggesting that nonlinear components of HP variability might be present even in connection with a small respiratory sinus arrhythmia (Porta et al., 2008). This apparent paradox dominates the field of the analysis of short HP variability series and deserves investigation.

We hypothesize that this apparent inconsistency could be explained by introducing in the analysis of short HP variability recordings the concept of multiple nonlinear components acting within a particular range of time scales.

The aim of this contribution is to stress the necessity of introducing the concept of temporal scales in the assessment of nonlinear dynamics in short HP variability recordings. Two complexity metrics assessing time irreversibility (Porta et al., 2008) and local prediction (Porta et al., 2007a) are considered in this study. The two methods cover different temporal scales: time irreversibility analysis focuses on very short time scales according to the reconstruction of the dynamics of the cardiac control in an embedding space of dimension equal to 2, while the local prediction method accounts for longer time scales by allowing the reconstruction of an optimal embedding space with larger embedding dimensions. These two methods will be applied to two different experimental protocols: healthy fetuses as a function of the gestational age (van Leeuwen et al., 2003; Lange et al., 2005) and healthy humans as a function of the magnitude of the orthostatic challenge (Montano et al., 1994; Porta et al., 2007b, 2011). The percentage of fetuses or adults exhibiting nonlinear dynamics detected according to a surrogate approach (Theiler et al., 1992; Schreiber and Schmitz, 2000) will be compared.

## Methods

### Irreversibility analysis

The series HP = {HP(*i*), *i* = 1,… *N*}, where *i* is the progressive cardiac beat counter and *N* is the series length, is said to be reversible if its statistical properties are invariant with respect to time reversal. Time irreversibility is incompatible with Gaussian linear dynamics produced by an autoregressive moving average process distorted by a static nonlinear invertible transformation modifying the distribution of the series (Weiss, 1975). Therefore, irreversibility analysis has been proposed to detect nonlinear dynamics (Weiss, 1975).

In the present study we applied the index proposed by Porta et al. (2008) assessing the percentage of negative first variations (NV%) in HP. More precisely, NV% computes the number of ΔHP(*i*) = HP(*i*+1)-HP(*i*) smaller than 0 divided by the number of ΔHP(*i*) different from 0 (multiplied by 100). When HP is reversible, NV% is about 50. A departure from 50 implies that NV% is different when the series is reversed in time (a negative variation becomes positive under time reversal), thus the series is irreversible and, necessarily, nonlinear (Porta et al., 2008). Since NV% is computed in an embedding space with dimension equal to 2 (Casali et al., 2008), this index focuses very short time scales.

### Local prediction based on K nearest neighbors

Given the series HP let us construct the set of patterns HP*_L_* = {HP*_L_*(*i*) = [HP(*i*),HP(*i*-1),…,HP(*i*-*L*+1)], *i* = *L*,…,*N*} reconstructed with the technique of the delayed embedding coordinates (Takens, 1981). Each pattern is actually a point in an embedding space with dimension *L*. Local prediction hypothesizes that there is a continuous function *f*(·), even nonlinear, linking past values, HP*_L_*(*i*), to the future sample, HP(*i*+1), such that, if two patterns, HP*_L_*(*i*) and HP*_L_*(*j*), are similar, their evolution HP(*i*+1) and HP(*j*+1) will be close (Farmer and Sidorowich, 1987). The local prediction approach based on *k* nearest neighbors assumes that the *k* nearest neighbors of the current pattern HP*_L_*(*i*), i.e., the HP(*j*) vectors, are helpful to predict its future evolution, HP(*i*+1). The best prediction of HP(*i*+1) was defined as the weighted mean of the evolutions of the *k* nearest neighbors, i.e., the HP(*j*+1) values, where each weight is the inverse of the distance between HP*_L_*(*i*) and each HP*_L_*(*j*) (Sugihara and May, 1990). We followed the suggestions reported in Porta et al. (2007a) to set *k* = 30 and to select the Euclidean norm to calculate the distance. The cost function utilized to assess prediction is the complement to 1 of the squared correlation coefficient between HP and its best prediction (Porta et al., 2007a). It is bounded between 0 (full predictability) and 1 (full unpredictability) and it exhibits a minimum over *L* when past values are fruitful to reduce the uncertainty about future values (Porta et al., 2007a). The pattern length *L* at the minimum (i.e., L_min_) was taken as the optimal amount of past samples helpful to predict future values and provided an estimate of the optimal embedding dimension (Porta et al., 2007a). The minimum, searched with *L* ranging from 1 to 12, was taken as unpredictability index (UPI). The cost function was evaluated in-sample (i.e., the predictor is evaluated over the same data utilized to set it) and the self-exclusion of HP*_L_*(*i*) from the set of neighbors of HP*_L_*(*i*) was utilized to circumvent the contribution of the reference vector to the prediction (Theiler, 1986).

### Surrogate approach

We utilized a surrogate approach to check for the presence of nonlinear components (Theiler et al., 1992). We set as a null hypothesis that the series is a linear Gaussian process eventually distorted via a nonlinear static invertible transformation. This null hypothesis is helpful for detecting dynamical nonlinearities using both NV% and UPI as a discriminating statistic. Accordingly, we built surrogate series with the same second-order statistical properties (i.e., with preserved power spectrum) and the same distribution (i.e., with preserved histogram) as the original ones, thus maintaining linear components of HP series and static nonlinearities eventually distorting the HP distribution. Iteratively-refined amplitude-adjusted Fourier transform surrogates were constructed according to Schreiber and Schmitz (2000). The maximum number of iterations was set to 100. We constructed a set of 250 surrogates for each original sequence. The parameters NV% and UPI were calculated over the surrogate series (NV%_s_ and UPI_s_) and over the original series (NV%_o_ and UPI_o_). If NV%_o_ was smaller than the 2.5th percentile of the NV%_s_ distribution or larger than the 97.5th percentile, the null hypothesis of reversibility was rejected and the original series was said to be irreversible, and according to Weiss (1975), to be nonlinear. If UPI_*o*_ was smaller than the 5th percentile of the UPI_s_ distribution, the null hypothesis of linearity was rejected (i.e., the original series was predicted better than surrogates) and the original series was said to be nonlinear.

## Experimental protocol and data analysis

### Experimental protocol

The data belong to two historical databases designed to evaluate: (1) in healthy fetuses the progression of the maturation of the autonomic nervous system (van Leeuwen et al., 2003; Lange et al., 2005); (2) in healthy humans the physiological adjustments during a graded orthostatic challenge (Porta et al., 2007b). We make reference to those studies for a detailed description of the population and experimental setup.

The first database was composed of 66 fetal magnetocardiographic recordings from 22 healthy fetuses in singleton pregnancies. Sampling rate was 1 kHz. The fetuses underwent recordings of 5 min with mother at rest between the 16th and the 40th week of gestation (WoG). All 22 fetuses had three recordings, one per period of gestation (PoG) according to the following definitions: (i) PoG1: from 16th to 24th WoG; (ii) PoG2: from 25th to 32nd WoG; (iii) PoG3: from 33rd to 40th WoG. As reported in van Leeuwen et al. (2003) the protocol adheres to the principles of the Declaration of Helsinki and was approved by the local ethical review board. Written informed consent was obtained from all pregnant women.

The second database was composed of surface electrocardiogram recordings (II lead, sampling rate was 1 kHz) from 17 healthy humans (aged 21–54, median = 28; 7 females and 10 males) at rest (R) in supine position and during head-up tilt (T). After 7 min at R, the subjects underwent a session (lasting 10 min) of T with table angle randomly chosen within the set {15,30,45,60,75,90} (T15, T30, T45, T60, T75, T90). Each T session was always preceded by an R session and followed by 3 min of recovery. The subjects underwent all T sessions without experiencing presyncope signs. The analyses were performed after about 2 min from the start of the T maneuver. As reported in Porta et al. (2007b) the protocol adheres to the principles of the Declaration of Helsinki and was approved by the local ethical review board. Written informed consent was obtained from all subjects.

The data are available from the corresponding author upon request.

### Data analysis

HP was approximated as the time distance between two consecutive R-wave peaks detected on the surface electrocardiogram. In the prenatal protocol fetal R-wave peaks were identified in the channel with the highest signal-to-noise ratio using a template matching approach after the digital subtraction of the maternal component. The time resolution of the fetal HP was 1 ms. In the protocol relevant to healthy individuals undergoing the *T*-test, R-wave peaks were detected using a traditional method based on a threshold on the first derivative. The jitters in locating the R-wave peak were minimized using parabolic interpolation, thus achieving a time resolution smaller than 1 ms. All R-wave peak detections were carefully checked to avoid erroneous identifications or missed beats. The missed R-wave peaks were manually inserted and the erroneous detections were fixed. In case of the presence of non-sinus beats cubic spline interpolation technique was applied over those HP values that were directly influenced by the occurrence of the non-sinus beats. Few corrections were made and the percentage of the corrections was substantially below 5%. Sequences of exactly 256 values were utilized because this choice favored the application of the fast Fourier transformation, thus speeding up surrogate generation. The length of the sequence was in keeping with suggestions provided by standards on short-term analysis of HP variability (Task Force, 1996). The analysis was carried out in the beat-to-beat domain to avoid reinterpolation that artificially increases linear correlation in relation to the adopted reinterpolation rate, and eventually blurs nonlinear dynamics (Theiler, 1986). The sequences were randomly chosen inside the reference periods. Since the adopted methods require stationarity, attention was paid to avoid strong nonstationaries. As a consequence, if the random selection picked up a sequence with evident nonstationarities such as slow drifting of the mean or sudden changes of the variance according to the test proposed in Magagnin et al. (2011), the sequence was discarded and a new random selection was performed until a stationary sequence was detected. The percentages of subjects exhibiting nonlinear dynamics were calculated in both databases and indicated as NL%.

### Statistical analysis

The normality of the distributions of the HP series was tested by the Kolmogorov-Smirnov test. Repeated measures one way analysis of variance (Dunnett's test for multiple comparisons), or Friedman repeated measures analysis of variance on ranks (Dunnett's method for multiple comparisons) when appropriate, was applied to check the significance of the differences between the experimental conditions and the control one (i.e., PoG1 in the case of the protocol on fetuses and R in the case of T protocol). Pearson product moment correlation analysis, or Spearman rank order correlation analysis when appropriate, was utilized to assess the significance of the correlation of HP mean and variance on WoG or tilt table angles. If L_min_ was larger than 2 in more than 95% of the fetuses or subjects in the assigned experimental condition, we concluded that L_min_ was significantly different from 2. The χ^2^ test was utilized to check the significance of the difference between the percentage of fetuses or subjects exhibiting nonlinear dynamics among the experimental conditions. Values of L_min_, HP mean and variance were reported as mean ± standard deviation. A *p* < 0.05 was considered significant.

## Results

### Time domain indexes

In the protocol on fetuses the HP mean was 404 ± 13, 421 ± 23 and 424 ± 23 ms during PoG1, PoG2, and PoG3 respectively with values during PoG2 and PoG3 significantly larger than those during PoG1. The HP variance was 49 ± 58, 203 ± 152, and 298 ± 392 ms^2^ during PoG1, PoG2, and PoG3 respectively with values during PoG2 and PoG3 significantly larger than those during PoG1. The HP mean and variance were significantly related to WoG (*r* = 0.364, *p* = 2.62^.^10^−3^ and *r* = 0.399, *p* = 9.16^.^10^−4^ respectively).

In the protocol on healthy subjects undergoing graded T the HP mean was 989 ± 109, 929 ± 116, 849 ± 83, 795 ± 85, 749 ± 89, 730 ± 85, and 732 ± 90 ms during R, T15, T30, T45, T60, T75, and T90 respectively. The HP mean during T30, T45, T60, T75, and T90 was significantly smaller than that at R. The HP variance was 4452 ± 3554, 4797 ± 3500, 3555 ± 2713, 2833 ± 1992, 2901 ± 2164, 2752 ± 2056, and 3039 ± 2886 ms^2^ during R, T15, T30, T45, T60, T75, and T90 respectively. The HP variance during T75 and T90 was significantly smaller than that at R. The HP mean and variance were significantly related to the tilt table angles (*r* = −0.687, *p* = 6.13^.^10^−18^ and *r* = −0.23, *p* = 1.18^.^10^−2^ respectively).

### Embedding dimension

NV% was derived from a bi-dimensional embedding space reconstruction of the HP dynamics (i.e., *L* = 2 beats). *L* = 2 beats corresponded to 0.81, 0.84, 0.85 s during PoG1, PoG2, and PoG3 respectively and to 1.98, 1.86, 1.70, 1.59, 1.50, 1.46, and 1.46 s during R, T15, T30, T45, T60, T75 and T90 respectively. UPI was derived in an optimized embedding space: the optimal embedding dimension, L_min_, was 4.0 ± 1.9, 2.9 ± 0.8, and 3.9 ± 1.6 beats during PoG1, PoG2, and PoG3 respectively and was 4.5 ± 1.4, 4.4 ± 1.7, 3.5 ± 0.7, 3.6 ± 1.4, 3.2 ± 0.8, 3.0 ± 0.4, and 3.4 ± 0.7 beats during R, T15, T30, T45, T60, T75, and T90 respectively. In the protocol on the fetuses the values of L_min_ were significantly different from *L* = 2 during PoG3: indeed, L_min_ was larger than 2 in more than 95% of the fetuses during PoG3. During the graded T protocol the values of L_min_ were significantly different from *L* = 2 in all experimental conditions. When the ranges of L_min_ were expressed in seconds, they became 1.64 ± 0.77, 1.21 ± 0.35, and 1.66 ± 0.68 s during PoG1, PoG2, and PoG3 respectively and 4.42 ± 1.36, 4.04 ± 1.54, 3.00 ± 0.61, 2.90 ± 1.09, 2.38 ± 0.61, 2.19 ± 0.26, and 2.50 ± 0.52 s during R, T15, T30, T45, T60, T75, and T90 respectively.

### Detection of nonlinear dynamics

**Figure 1:**
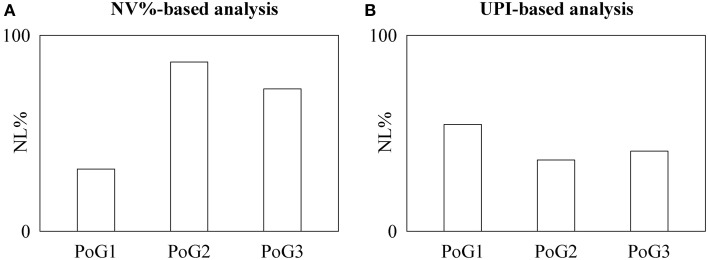
**The bargraphs show the percentage of fetuses exhibiting nonlinear dynamics, NL%, as detected by the NV%-based time irreversibility (A) and UPI-based local prediction (B) analyses as a function of the period of gestation (i.e., PoG1, PoG2, and PoG3)**.

In fetuses results obtained by time irreversibility analysis (Figure 1A) were completely different from those based on the local prediction approach (Figure 1B). Indeed, while NL%, as detected by time irreversibility analysis, increased as the pregnancy progressed, NL%, as detected by the local prediction approach remained constant. The χ^2^ test supported this observation: indeed, NL%, as detected by time irreversibility analysis, was significantly smaller in PoG1 than in the pooled set formed by PoG2 and PoG3.

**Figure 2:**
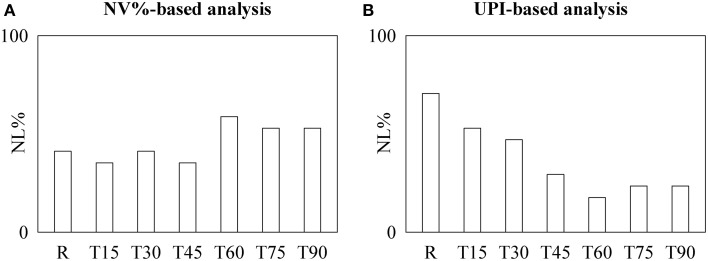
**The bargraphs show the percentage of healthy humans exhibiting nonlinear dynamics, NL%, as detected by the NV%-based time irreversibility (A) and UPI-based local prediction (B) analyses as a function of the tilt table inclination during graded *T*-test (i.e., R, T15, T30, T45, T60, T75, and T90)**.

Similarly to the protocol on fetuses, discordant conclusions can be drawn from the one on healthy humans (Figure 2). Indeed, NL% detected by time irreversibility analysis was high at R and remained stable with tilt table angles (Figure 2A), while NL% assessed by the local prediction approach decreased (Figure 2B). This observation was corroborated by the χ^2^ test: indeed, when the pooled set formed by R, T15, T30, and T45 was contrasted with that formed by T60, T75, and T90, we found a significant decrease of NL% as detected by the local prediction method.

## Discussion

The original findings of this study can be summarized as follows: (i) two distinct types of nonlinear dynamics can be detected in short HP variability series; (ii) these two distinctive features span different temporal scales and need embedding spaces of different dimensionality to be resolved.

### Comparison between different complexity metrics for the detection of nonlinear components in short HP variability recordings

When time irreversibility and local prediction analyses were applied to short HP variability series recorded in fetuses as a function of the WoG and during graded orthostatic challenge as a function of the tilt table inclination, opposite conclusions can be drawn. Indeed, in the protocol on fetuses NL% as detected by NV% increased as a function of the PoG, while NL% derived from UPI remained stable and in the protocol on healthy humans NL% as detected by UPI significantly decreased with tilt table inclination, while NL% as derived from NV% remained constant. It is worth noting that results derived from time irreversibility analysis do not depend on the choice of the low dimensional irreversibility index: indeed, indexes alternative to NV% such as those proposed by Guzik et al. (2006) and Ehlers et al. (1998) provided the same outcome. In addition, results obtained by the local prediction approach do not depend on the coarse graining procedure: indeed, different techniques (Kantz and Schreiber, 1997; Porta et al., 2000) leading to indexes alternative to UPI gave the same outcome. These findings are in agreement with the inconsistent conclusions present in literature (Kanters et al., 1996, 1997; Sugihara et al., 1996; Fortrat et al., 1997; Porta et al., 2000, 2007a, 2008). However, in spite of this evident paradox until now no study brought it to the attention of the scientific community and tried to provide a possible reconciliatory interpretation.

### Two distinct types of nonlinear dynamics are observable in short HP variability recordings

We propose the following explanation to the observed, apparently conflicting, findings. Time irreversibility analysis is based on a simple index (i.e., NV%) derived from a bi-dimensional embedding space reconstruction of the HP dynamics (i.e., *L* = 2 beats). This index allows the focalization of very short time scales: indeed, longer time scales are roughly unfolded in a bi-dimensional phase space and remain largely unresolved. On the contrary, local prediction method is based on a marker (i.e., UPI) derived from significantly higher dimensional embedding spaces (i.e., L_min_ > 2 beats). Therefore, longer temporal scales can be unfolded and more complex nonlinear features can be determined. As a consequence of the different time scale focalization, NV% and UPI might detect diverse nonlinear components of the HP dynamics, thus explaining the difference between NL% as derived from NV% and NL% as derived from UPI in both protocols. Given that the nonlinear components at longer time scales, as detected by UPI, were found more frequently in healthy individuals at low tilt table angles and nonlinear features at very short time scales, as detected by NV%, were found more frequently in fetuses in the late PoGs, it is difficult to associate these two different nonlinear components to a specific state of the autonomic nervous system. Indeed, since the parasympathetic regulation of the fetal heart becomes functional during PoG1 and earlier than the sympathetic control developing during PoG2 (Papp, 1988), results relevant to NV% in the protocol on fetuses might suggest an involvement of the sympathetic regulation in producing nonlinear HP dynamics but sympatho-vagal interactions cannot be excluded as well. Similarly inconclusive results about the role of a specific branch of the autonomic nervous system can be derived from the T protocol. Indeed, results relevant to UPI, indicating a more frequent presence of nonlinear features at the smallest tilt table angles when the sympathetic drive is lower (Montano et al., 1994; Cooke et al., 1999; Furlan et al., 2000; Porta et al., 2007b), might suggest an involvement of the vagal regulation in producing nonlinear HP dynamics but a possible role of the sympathetic control cannot be dismissed. Therefore, in both protocols the null hypothesis that nonlinear dynamics could be generated by the interactions of the two branches of the autonomic nervous system cannot be rejected.

It is worth noting that no association between nonlinear components and HP mean or variance was found. Indeed, while in the two protocols HP mean and variance exhibited opposite trends (i.e., HP mean and variance progressively increased in the protocol on fetuses and gradually decreased in the protocol on healthy humans), NL% behaved similarly (i.e., NL% tends to increase or decrease in both protocols) once the method for the detection of nonlinear dynamics was assigned.

## Limitations of the study and future developments

Even though the findings of the study have been discussed in terms of sympatho-vagal interactions, other factors (see Cohen and Taylor, 2002, for a review), known to influence HP variability and the action of which is compatible with the time scales that can be resolved using short HP variability recordings, can play a significant role. We advocate upcoming studies to characterize further the two identified nonlinear components.

Since age and gender influences HP dynamics (Ryan et al., 1994; Voss et al., 2012; Catai et al., 2014) and its interactions with other cardiovascular variables, such as respiration (Nemati et al., 2013; Porta et al., 2014), these factors can play a role in governing the presence of the observed types of nonlinear components. Future studies should specifically address this issue by keeping separated the two identified nonlinear components.

## Conclusions

The contemporaneous application of two different metrics for the assessment of nonlinear dynamics over the same datasets allows the detection of two distinct types of nonlinear dynamics in short HP variability recordings. These distinctive types span different temporal scales but their link to the state of the autonomic nervous system remains elusive. However, accounting for the presence of these two diverse types of nonlinear components is necessary for interpreting the apparent contradictory findings present in literature. In addition, these findings stress the need to introduce a multiscale approach in the analysis of nonlinear components of HP dynamics.

## Author contributions

AP contributed to the conception and design of the work, interpretation of the data, drafting the article, critical revision of the manuscript, final approval of the version to be published. VB, AM, and BM. contributed to the analysis of the data, interpretation of the data, critical revision of the manuscript, final approval of the version to be published. DC, PL, TG-R, AT, and AC contributed to the acquisition of the data, interpretation of the data, critical revision of the manuscript, final approval of the version to be published.

### Conflict of interest statement

The authors declare that the research was conducted in the absence of any commercial or financial relationships that could be construed as a potential conflict of interest.

## Abbreviations

HP: heart period
NV%: percentage of negative first variations
L_min_: optimal embedding dimension
UPI: unpredictability index, NL%, percentage of fetuses or adults exhibiting nonlinear HP dynamics
PoG: period of gestation
WoG: week of gestation
R: rest condition in supine position
T: head-up tilt.
